# Systems Thinking in Mental Health Patient Safety: A Narrative Review of Complex Adaptive Systems

**DOI:** 10.1111/jep.70080

**Published:** 2025-05-19

**Authors:** Alexander Challinor, Oladayo Bifarin, Esmaeil Khedmati Morasae, Pooja Saini, Kathryn Berzins, Rajan Nathan

**Affiliations:** ^1^ Mersey Care NHS Foundation Trust Liverpool UK; ^2^ Institute of Population Health University of Liverpool Liverpool UK; ^3^ National Institute for Health and Care Research Applied Research Collaboration North West Coast Liverpool UK; ^4^ Faculty of Health Liverpool John Moores University Liverpool UK; ^5^ National Institute for Health and Care Research (NIHR) London UK; ^6^ School of Management University of Bradford Bradford UK; ^7^ Health Technology Assessment Unit, Applied Health Research Hub University of Central Lancashire Preston Lancashire UK; ^8^ Faculty of Health, Medicine and Society, Chester Medical School University of Chester Chester UK; ^9^ Cheshire and Wirral Partnership NHS Foundation Trust Chester UK

**Keywords:** complex, healthcare, mental health, patient safety, system

## Abstract

Despite the growth of knowledge and interest into safety and quality in healthcare more generally, the exploration in mental healthcare has been deemed to be in a narrow isolated ‘world of its own’. It is possible that relatively little attention is being paid to the processes and interdependencies within the mental health patient safety system. This may result in simplistic static measures of what the system/organisation *has*, not what it does (or doesn't do). This can limit the potential for learning and affecting change. To investigate systems thinking in mental health patient safety, we conducted a narrative review into the extent of evidence streams supporting systems and complexity thinking approaches. We sourced a total of 89 reports for analysis with six themes identified. These themes included studies evaluating patient safety events that have occurred within mental healthcare, research that has investigated components of the safety system, and studies that have investigated how patient safety incidents are responded to, investigated, and learned from. The review evaluated the use of systems thinking and complexity research in patient safety, and research encapsulating patient and carer involvement. Most research has focused on the analysis of historic approaches to incident investigation and on system‐based factors of patient safety, with little attention being paid to systems and complexity thinking approaches. The relationships between components were often ignored in the non‐systemic studies sourced, with relationships between components not investigated and unknown. With policymakers recommending changes in patient safety practice through system‐based approaches, it is important that its implementation is evaluated robustly with consideration of the multiple levels of the healthcare system. Future research should aim to incorporate systems‐thinking approaches to model the safety system, and to improve our understanding of the highly interconnected technical and social entities that dynamically produce emergent behaviour across the system.

## 1 Introduction

Healthcare providers and systems expend a great deal of effort and resource on improving patient safety, the goal of which is to reduce the occurrence of avoidable harm [1]. The patient safety system is in itself a discipline, with the goal of achieving a trustworthy system of health care delivery and a system that reduces the incidence and impact of avoidable harm [2]. Its importance emphasised by the evidence that adverse events are often avoidable and widespread, where patient safety demands a system designed to be reliable and resilient when faced with the complexity and unpredictability of healthcare [3]. Effective prevention of harm positively impacts health outcomes, improves system efficiency, fosters trust in staff, patients and communities and reduces costs related to patient harm [1, 4].

Understanding how the patient safety discipline operates as a whole system builds an understanding of how the independent, interacting parts of the system work together to achieve the goal of improving safety outcomes [5]. A whole system approach is generally defined as a method of understanding complex issues by studying the interconnected parts and relationships within the system [6]. More specifically, systems thinking focusses on the inter‐relationships, the dependencies, and interactions of a system, reflecting the system's boundaries [7]. Complexity science evaluates the dynamic, unpredictable, adaptive elements within the system, where combined elements of systems and complexity thinking may conceptualise a health system as a complex adaptive system [7].

Healthcare involves a highly connected system of individuals (staff, patient, service users, carers, relatives etc.,), teams, processes, equipment, infrastructure, and institutions that work together [8]. The functioning within this complex system relies on the continuous use of adaptive capacities to cope with the variability in the system, where safety is an emergent property of relations and interactions between actors and elements in the system [9, 10]. This is what the system *does*, not what the system has [10, 11]. This is aligned with the Safety‐II perspective, where it brings the presence of safety into focus, rather than determining safety by its absence (e.g., through frequency of adverse incidents) [12]. The view of safety as the absence of incidents alongside a presumption that things go wrong due to an identifiable failure in specific component(s) of a system is termed Safety‐I [13].

The Safety‐I approach alone has its limitations. The system's performance and behaviour shift over time and cannot be fully understood by knowing its individual components or by viewing it through linear cause‐effect relationships [14]. The Safety‐II perspective recognises this complexity and inherent variability and by combining the two ways of thinking, healthcare can facilitate everyday work effectively and safely whilst maintaining the adaptive capacity to respond to and learn from any inevitable surprises [13]. Contemporary safety science, general healthcare patient safety research, and the National Health Service (NHS), United Kingdom, safety strategies have acknowledged the importance of system‐based practice [15, 16].

An aspect of the patient safety system that has recently adopted a systems‐based approach is safety incident investigation. The NHS has recognised the need for change, advocating for ‘strong/effective systems‐based improvements to prevent or significantly reduce the risk of a repeat incident’ [15, 17]. It highlights the need for ‘insight’, an approach to improve understanding of safety by drawing from multiple sources of patient safety information and supports a system‐based approach to learning from patient safety incidents. This systems approach to incident investigation should aim to answer fundamental questions about the wider system in which people operate, the opportunities for risk, and to develop strategies designed to improve quality of care and mitigate harm [8]. This change from preventable harm and causality is a welcomed approach. However, the patient safety strategies used to address errors, and the learning/improvements made to reduce poor outcomes are likely to be flawed if we have a limited understanding of what the patient safety system *does*, how it succeeds but sometimes fails.

Research considering a whole systems‐thinking may take the form of different empirical approaches. For example, a study may evaluate the safety system through the development of a systems dynamic map that can model and analyse the interacting components. Research may also investigate the purpose and values of the patient safety system (e.g., incident investigation, safety events, harm outcomes), system‐based considerations such as culture, and leadership, and whether the system promotes innovation, adaptation and learning.

There are likely to be benefits if patient safety can be studied and understood through the lens of a whole complex system. This perspective can lead to an understanding of its dynamic behaviour and potentially steer it towards more favourable directions and outcomes [6]. It is possible that relatively little attention is being paid to the processes and interdependencies within the mental health patient safety system, resulting in simplistic static measures of what the system/organisation *has*, not what it does (or doesn't do), limiting the potential for learning and affecting change. To our knowledge, little research has focused on the concepts required for system level reform in mental health services, internationally and in the United Kingdom.

Despite the growth of knowledge and interest into safety and quality in healthcare more generally, the exploration into safety in mental healthcare has been deemed to be in a narrow isolated ‘world of its own’ [18]. The empirical evidence base for patient safety in mental health services is significantly under‐researched in comparison to other nonmental health settings [19]. Mental health research is yet to embrace this view of patient safety in the strategic actions to reduce preventable harm [10]. The assumption that applying learning from general health care to mental health settings is likely to be a flawed one. There are specific challenges of safety within mental healthcare that complicate the system. Arguably, health service design rarely incorporates real world demand modelling, but is quick to rely primarily on consensus and some retrospective data. This tends to manifest in form of epistemic uncertainty, difficulties in incorporating patient's feedback into redesigning systems of care, and the nature of mental illness and risk. The behaviours associated with serious mental illness (e.g., self‐harm and/or violence to others) and the interventions aimed to manage these (e.g., risk assessment tools, restraint) add further layers of complexity to the concept of patient safety.

Achieving parity with general healthcare research will require accumulation of a robust body of evidence that provides a systems‐level understanding of quality of care and patient safety in mental health settings. Reviewing the current evidence‐base for systems‐based considerations in mental health patient safety would provide a good foundation on which to build from.

## 2 Aims

The aim of this study was to review the evidence streams supporting a complex system approach to patient safety in mental healthcare. The focus of the narrative review was to explore how systems‐thinking approaches have been applied to patient safety in mental healthcare settings.

## 3 Methods

We used an exploratory narrative methodology to review the evidence investigating patient safety in mental healthcare [20]. The available literature was reviewed using three databases—Embase, CINAHL, Psychinfo. More than one database was chosen to ensure sufficient coverage. Specialised databases of CINAHL and Psychinfo were chosen as these databases more closely align with the review topics.

The following keywords were used:

− Patient safety
− AND (psychiatry, mental health, mental illness, mental disease or mental disorder)
− AND (system or systems).

The snowball method was used with backward citation tracking applied on articles that were included in the study. Grey literature was sourced through the snowball method and a purposive search of King's Fund and Department of Health electronic libraries. The literature search was conducted in February 2024, with no limitation placed on date or publication type.

### 3.1 Eligibility Criteria

We focussed on research articles and reports that considered patient safety within the system of mental healthcare. A screen of identified studies reviewed whether initial criteria were met, including whether the study was conducted in a mental health setting and whether it investigated patient safety. We did not place any restrictions on the basis of study design or publication status, for example systematic reviews or ‘grey’ literature. We excluded non‐English language publications.

Articles were purposively selected for inclusion and exclusion according to their conceptual contribution to the research and literature based on our aims. Data extraction included descriptions of publication date, study context, aims, study design, and the methodology used. The conceptualisation of the studies focus on ‘systems’ were extracted. The findings were synthesised narratively, through development of textual descriptions of studies, and then through identification of common themes that emerged from the narrative review. Key conceptual themes were chosen and described in more detail. The narrative review was designed and conducted according to guidelines for the quality assessment of narrative review articles [20].

### 3.2 Patient and Public Involvement

This paper formed part of a larger NIHR funded research project aimed at understanding more about the patient safety system in mental healthcare. We worked with patient and public involvement (PPI) groups in the design of the study. The PPI groups were used to consider potential themes that may emerge from the narrative review. This ensured that patient, public and carers perspective was captured in the outcomes from the data.

## 4 Results

**Figure 1:**
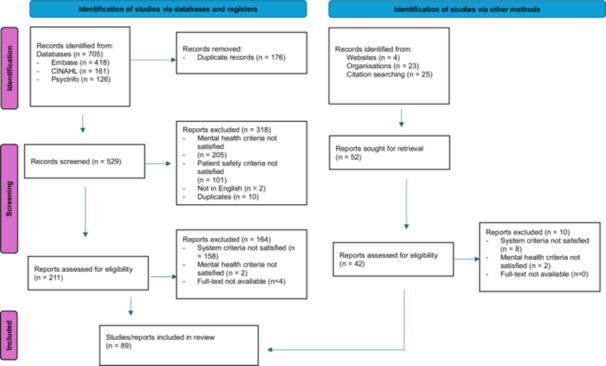
Flow diagram of studies identified from databases, registers and other sources. Adapted from Page et al. [21].

The search revealed 705 records (Figure 1), which reduced to 529 after duplication. Articles were screened by author AC and consensus discussions were conducted with author RN where exclusion/inclusion criteria were uncertain to have been met. The initial screen revealed 211 studies that had conducted patient safety research in mental healthcare with reference to a system. These studies were further assessed for eligibility. Figure 1 shows the studies identified and screen.

### 4.1 Description of General Characteristics of Included Sources

A total of 89 sources were chosen for analysis, which included empirical studies, reports and policy documents. Patient safety was examined across mental healthcare, within a range of different care settings including inpatient general adult mental healthcare, secure mental healthcare, community mental healthcare, and veteran healthcare, either in single sites or in more than one setting. Of those 89 sources, 62 were research studies, 16 were policy documents/guidance and 11 were reports from an organisation. The policy documents were largely NHS documents on patient safety strategies. Of those that were a research publication, 10 (*n* = 10) were editorials exploring the safety system. The remaining 52 research studies consisted qualitative (*n* = 23), quantitative (*n* = 6) and mixed‐method (quantitative and qualitative) (*n* = 12) studies designs. There were 11 reviews conducted (*n* = 11).

### 4.2 Patient Safety in the Mental Healthcare System

Six themes of patient safety in the mental healthcare system were identified from different conceptualisations of systems extracted from the sourced literature. These included; studies evaluating patient safety events that have occurred within mental healthcare; research that has investigated empirically defined system‐based components of patient safety; the investigation, response and learning from adverse incident investigation; and whether systems‐thinking or a systems‐based methodological approach had been applied to the safety system.

A final theme was research encapsulating patient and carer involvement, which was guided from the PPI group involvement and the focus on patient/carer involvement in the NHS safety policies [15].

Theme 1 Conceptualising patient safety events within the mental healthcare system

Several studies have attempted to better define patient safety within inpatient mental healthcare defining categories of safety concerns that may arise [19, 22, 23, 24]. These studies encapsulate key research topics but don't necessarily capture system‐based considerations or conceptualise the topics within the scope of system‐based practice.

A systematic review of patient safety in the inpatient mental healthcare setting delineated several categories of patient safety research, noting that they found limited research in systems level improvement [19]. Of those that have evaluated system‐based practices, the studies sought to understand the type of errors and the contextual factors that precipitate them, with consideration given to systems level improvement.

Particular areas of focus were on medication errors [25], self‐harm/suicide [26, 27, 28], falls [29] and violence/aggression to others [30]. Three studies reviewed medication errors, one during an inpatient stay [31], one following discharge from hospital [32], and one in the community setting [33]. Two of those studies advocated a systems‐based approach to reviewing incidents and one study applied a systems‐based analysis to the medication errors [33]. Research into deaths by suicide in the veterans' health administration revealed limitations in their systems of care, in particular poor communication between components of the system that contribute to patient safety [26].

Theme 2 System‐based considerations within patient safety research in mental health

System‐based considerations described within NHS and Department of Health and Social Care guidance documents detail components (e.g., technology, task complexity, culture, stress, fatigue, leadership and management, policies, workplace design) of the patient safety system [34]. These components are thought to be system‐based considerations where these factors have been studied within patient safety research. Our narrative review found studies that have examined these factors. However, research has not consistently conceptualised them as system‐based components or factors in the patient safety system.

Most studies directly examining system‐based considerations have done so via qualitative investigation of workplace/organisational culture. Patient safety culture has been found to be a key aspect of the NHS patient safety strategy [16]. We found seven studies whose primary objective was to explore patient safety culture in mental healthcare [35, 36, 37, 38, 39, 40, 41]. Other studies that have featured patient safety culture have examined nursing skills [42], nursing interventions [43], well‐being and burnout [44, 45], work patterns and fatigue [46], mental health professionals' perspectives on safety [47, 48, 49, 50], patient experiences [51] and safety in secure mental healthcare [52, 53, 54, 55].

Two studies implemented a Team Strategies and Tools to Enhance Performance and Patient Safety program (TeamSTEPPS) to systematically weave safety through the organisation and hospital culture, and to improve teamwork and communication [56, 57]. Two studies identified actions supporting patient safety culture around serious incident management frameworks [58, 59] and one study explored impact, nature and causes of medical errors [23]. Another study that examined incidents of aggression/violence revealed workplace environment and culture as critical patient safety components [30].

Alongside culture, one study discovered other system‐based factors including service processes, staff workload and communication systems [50]. A qualitative exploration of agitation, de‐escalation and restraints in a child and adolescent mental health hospital revealed that tasks, environmental factors, and organisation factors (connections between unit teamwork, communication and culture) were critical system‐based factors that can help reduce physical restraint use [60]. Another study used large‐scale simulation to improve safety regarding on‐site hospital emergencies revealing deficits in exchanging information and leadership [61].

One study used qualitative methods to investigate workforce characteristics and the quality of care in mental health services, demonstrating the impact of staff and skill mix on patient safety [62]. Other workforce considerations have included nursing staff numbers on conflict and containment [63]. Additionally, the ‘Safewards Model’ investigated both local and systematic factors (physical environment, regulatory frameworks, staff structure) to reduce conflict and containment rates on acute mental health wards [64, 65]. On a retrospective review of barriers and enablers on a ‘Safewards Model’ (interventions to prevent conflict and containment) the authors described healthcare system influences on its implementation. This included staffing, ward acuity, support and resources, preparation/training, ward climate, policies and leadership [66].

The NHS patient safety strategy details organisational factors as system‐based components [15]. With a focus on patient suicide, before‐and‐after analyses shows that service changes (staff training, policy/guidance implementation) and organisational factors (non‐medical staff turnover, incident reporting) were associated with reduced suicide rates [67]. One study evaluated the organisational risk strategies, policies and procedures in acute mental health trusts in a geographical region of the NHS [68]. This study found limited guidance in the promotion of good practice and barriers to the success of risk management in patient safety [68].

Theme 3 How patient safety incidents are responded to and investigated

The third theme within the review identified studies analysing how patient safety incidents are investigated. There is research evaluating how incidents were responded to and investigated historically, alternative approaches to incident investigation and studies exploring a system‐based approach to incident investigation.

An exploration of the how and when patient safety investigations should be investigated identified significant issues with the ‘Serious Incident Framework’ approach to incident investigations, including inappropriate use of the framework, poor quality of the reports, and a lack of learning and improvements to prevent recurrence of harm [69]. Research studies have critically analysed different approaches to investigating patient safety incidents in healthcare. Studies have evaluated the root cause analysis approach to the investigation of serious incidents in mental healthcare [70, 71, 72, 73]. The criticisms of the serious incident framework and root cause analysis shifted the NHS patient safety strategies focus onto system‐based analysis of investigations [34].

One study attempted to address patient safety concerns using a novel approach to identify case‐based putative causal factors that may lead to risk events [74]. The authors concluded that this hazard and operability approach can uncover causal factors amenable to change that may not be identified in single case investigations [74]. We found one study that evaluated a system‐based approach to the analysis of incidents in a mental healthcare setting [33]. This study reviewed risk factors associated with medication errors in community mental healthcare using the System Engineering Imitative for Patient Safety (SEIPS) framework identifying vulnerabilities in the system [33]. Additionally, objectives were found aimed at identifying learning from the incident and development of sustainable quality improvement strategies to improve patient safety [33].

With the traditional focus on how to investigate incidents and events that have occurred, an alternative approach is to evaluate and analyse real‐world performance variability and resilience within the healthcare system. We found one study that analysed a mental health service's day‐to‐day performance variability of discharge from inpatient care [75]. The study showed limitations in patient safety incident reporting systems and how using alternative methodologies healthcare management can better consider how to optimise and use resources [75].

Theme 4 Learning from patient safety incidents

Our narrative review found minimal exploration of the link between an investigation and the process of implementing evidence‐based improvements to reduce harm. We found two studies that used qualitative methodology to explore whether staff perceived incident reporting as having a positive effect on safety [76], and how health professionals use incident data to improve patient safety [77]. The first study showed that within mental healthcare the clinicians were less likely to use the reporting system and were more sceptical of its value [76]. The second study conducted interviews with staff and observations of incident review meetings, finding that very little consideration was given to system aspects of patient safety [77]. Both studies did not investigate whether the learning or approaches led to a reduction in harm. A study assessing recommendations from incident reports aimed at enhancing safety found that the most prevalent suggestion was that issues ‘should be discussed’, with minimal emphasis on system‐based components [30]. We found no studies that examined how systems approach to investigation may yield learning for the patient safety system or the organisation in mental healthcare.

Theme 5 A whole systems approach to patient safety

Ensuring a systems approach and systems thinking has been found to be a core theme within NHS patient safety initiatives [16]. As detailed above in theme 2, system‐based considerations have been investigated, although few studies have conceptualised their investigation within the scope of system‐based practice. Within those non‐systemic studies, the relationships between components were ignored, with relationships between components unknown. A whole systems approach would evaluate this, and the narrative review explored whether systems thinking, and complexity science had been applied to the patient safety system.

The need for adopting a systems‐level approach to patient safety is well‐documented within patient safety strategies and the empirical literature [16, 78, 79, 80, 81, 82, 83, 84]. One study used qualitative methodology to identify patient safety issues in mental healthcare [55]. These safety issues were then mapped onto the Yorkshire Contributory Factors Framework, a tool commonly used to general healthcare setting to provide a framework of factors that can contribute to patient safety incidents [85]. This approach recognises a system‐based exploration of the safety system [85].

We found no studies that used system dynamics modelling (SDM) to map the patient safety system. One study mapped out the role, benefits, challenges, and future directions of using SDM for suicide prevention [80], and four studies used SDM as an analytical tool to shape suicide prevention [21, 86], to understand the interpersonal theory of suicide in adults [87] and adolescents [88]. We found no studies that used a collaborative systems approach to model the patient safety system through the participation of patients, family and carers.

We note an ongoing organisational ethnographic approach aiming to understand how people involved with patient safety incidents are experiencing the newly implemented NHS patient safety policy [89]. An output from this will be a dynamic logic model that is revised throughout the research. It is unclear at this stage whether there will be consideration of patient safety as a complex adaptive system. An organisational ethnographic methodology would likely miss the benefits of systems mapping. This may include numerical figures, quantitative simulation and the provision of a testbed for intervention scenarios.

Theme 6 Patient/carer perspective on patient safety in mental healthcare

The PPIE work highlighted the theme of how the patient safety system incorporates patients and carers perspectives, within the system more generally and within the process of safety incident investigations. Evidence highlights the importance of involving staff, patient and families in incident investigations in an effective and compassionate way [34]. The NHS patient safety strategy encompasses this as a high‐level objective [15].

Our narrative review found several studies that included patients/carers in their study. Of those studies the majority were of a qualitative methodology. Other studies explored healthcare professionals' opinions regarding the role that mental health patients might play in patient safety [90]. Studies have used cross‐sectional surveys, interviews and focus groups to delineate patient safety priorities, system concerns and improvements, and safety issues [55, 91, 92]. Broader explorations of mental healthcare experiences reveal that safety is a vital factor in the relationship between the patient and service provider [90, 93, 94].

One study explored patient and carer perspective and involvement in patient safety research in mental healthcare [95]. The conclusion from the study was that although patients have been involved in a diverse array of safety research, most were in research focussed on restrictive practices and there were limitations in how patients were involved. They concluded that there needs to be improvements in embracing patient involvement to meaningfully improve the patient safety system [95].

## 5 Discussion

### 5.1 Main Findings

**Figure 2:**
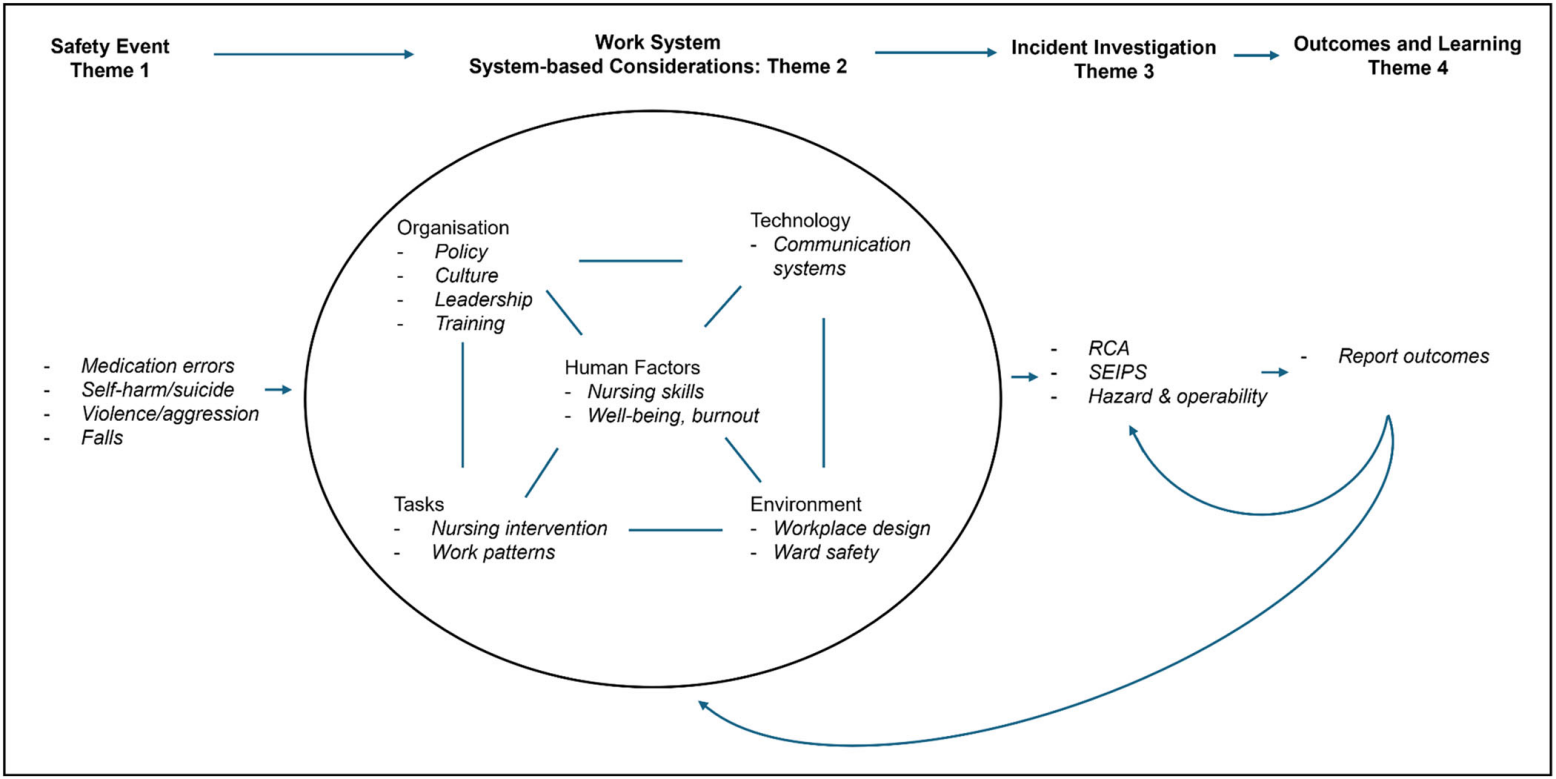
Adapted SEIPS model demonstrating the themes obtained from the narrative review. The text in *italics* notes what research has been conducted in these categories.

To the best of the authors' knowledge, this is the first study to identify and demonstrate the extent of available evidence supporting a complex system approach to patient safety in mental healthcare. This review identified several key issues relating to patient safety research that had considered system‐based practice. This includes conceptualising patient safety events and concerns within the system, the investigation of system‐based factors, investigating and learning from incidents, the use of complex adaptive system methodology within patient safety research, and the perspective of patient/carers. Most research has focused on the analysis of historic approaches to incident investigation and on system‐based factors of patient safety, primarily through the examination of safety culture as an emerging property of the system. Figure 2 provides a conceptual model of the main findings of the narrative synthesis. The model places the review literature into an adapted SEIPS model, demonstrating what has been studied empirically and where there are gaps in our understanding [33].

The review found that little attention has been paid to understanding the interdependencies within the system and how they may contribute to patient safety. Additionally, most of the research has not been conceptualised within the remit of (i) patient safety, and/or (ii) system‐based practice or thinking. This results in conceptual challenges in defining safety across the mental healthcare system as a whole and as a complex dynamic system. The results show that little attention is being paid to the processes and interdependencies within the patient safety system, potentially resulting in established silos of working with a small number of studies evaluating how the system promotes learning and affects change. There is an urgent need to cultivate a data‐driven culture by prioritizing the needs and concerns of those within the system.

These findings are similar to those found in other reviews, recognising the disparate bodies of research and the need for closer collaboration between mental healthcare and the wider field of patient safety [10, 18, 96]. The slower update of contemporary safety science into patient safety in mental healthcare is demonstrated in our review, where a large focus has been on isolated contributory factors/components of the patient safety system. Mental healthcare research is largely yet to explore safety outside of linear cause‐effect models when constructing the notion of preventable harm [10].

A substantial body of the empirical evidence base in mental healthcare (e.g., violence/aggression, self‐harm/suicide) does not directly align itself with patient safety theory, which may impact on the advancements of patient safety research, understanding the complexity of the system, and on the analysis and learning from incident investigation. One of these fields have recognised the role of system thinking and systems modelling, noting that its update within the field of mental health has been slow [80, 86, 87]. Although no studies have attempted to use SDM on the patient safety system, studies have begun to investigate system modelling within suicide prevention [80, 86, 87].

### 5.2 Implications for Patient Safety

It is encouraging that there is an emerging empirical evidence base for system‐based components in mental healthcare. This study will aid our understanding of the interaction of humans and other elements of the system to optimise staff, patient and carer wellbeing and improve system performance [6]. What we continue to lack is a greater understanding of the highly interconnected technical and social entities that dynamically produce emergent behaviour within the patient safety system.

Within healthcare, it is challenging to deconstruct patient safety incidents into linear cause‐effect sequences, where the system has inherent complexity, uncertainty and unpredictability [17]. This is compounded further with the conceptualisation of risk in mental healthcare. Despite this complexity, our narrative review and existing literature has shown that patient safety is studied within linear cause‐effect models [10]. A resultant outcome that has stemmed from the criticisms of previous incident response frameworks and through the incorporation of contemporary safety science into practice is the shift in the NHS patient safety strategies [15]. The emergence of the Patient Safety Incident Response Framework (PSIRF) prompts the consideration of a systems‐based approach to incident investigation where the SEIPS model is the framework chosen for investigating incidents [34]. This narrative review found only one study that evaluated the SEIPS approach to incident investigation in mental healthcare [33]. Future studies investigating the systems‐based approach to incident investigation and its outcomes are clearly required.

Our review also found minimal exploration of the link between incident investigation and the process of implementing evidence‐based improvements to reduce harm. Implementing a system‐based approach to investigating incidents may not address all the factors that interfere with the operationalisation of learning for everyday practice. For example, an investigation into a patient safety incident will interface on one hand with the service where the patient incident occurred (i.e., in direct contact with patients) and on the other hand through other people and forces, which may be removed in time and space from direct care but nonetheless affect how the care is delivered. A greater empirical understanding of the interdependencies and process across levels are needed to fully maximise the impact of the NHS patient safety frameworks and to optimise learning. A better understanding of how risk and safety is operationalised within real world complex healthcare systems will not only inform improvements in patient safety, but it will also mean that unintended consequences for healthcare professionals, patient and carers can be identified with a view to mitigating them.

### 5.3 Strengths and Limitations

This narrative review provides a comprehensive overview of systems approaches to patient safety system in mental healthcare. The search itself incorporated three databases, a review of the grey literature and searched reference lists. It was limited to studies in English with the grey literature sourced mostly applicable to UK healthcare practices. This can limit its generalisability to other settings. Another limitation is the use of one reviewer when screening and reviewing the sources, as well as a narrative rather than systematic approach. The aim was to explore a complex and broad topic bringing together the literature through a critical and interpretive lens. Consequently, this may offer interpretations and conclusions drawn from the review. Nevertheless, the review identifies gaps in the literature and considers future approaches for research and clinical practice.

## 6 Conclusion

The vast majority of patient safety research and systems‐based research has been conducted outside of mental healthcare. There are encouraging signs of an emerging research investigating system‐wide components in patient safety in mental healthcare. The shift in the NHS patient safety strategy to ensure a system‐based focus to incident investigation is also promising. There is hope that this will promote further research on system‐based components conceptualised within the field of patient safety research and on a whole systems approach to patient safety. To advance the field, the patient safety as a complex adaptive system must be acknowledged, and the systems interdependencies and processes must be considered. Further research and meaningful collaborative approaches with professionals, stakeholders, patients and carers are needed to unpack the dynamic complexity of the patient safety system.

## Conflicts of Interest

Oladayo Bifarin is a National Institute for Health and Care Research Leader. Kathryn Berzins is funded by NIHR ARC NWC. The views expressed in this article are those of the author(s) and not necessarily those of NIHR or the Department of Health and Social Care.

## Data Availability

Permission to access our study data is only granted to researchers in the study team.
